# Effect of Budesonide Nasal Irrigation in Patients With Chronic Rhinosinusitis With Nasal Polyps Without Prior Sinus Surgery: A Randomized, Double‐Blind, Placebo‐Controlled Study

**DOI:** 10.1002/alr.70112

**Published:** 2026-02-13

**Authors:** Juliana Sant'Ana, Isabela Pontes, Caio Floriano, Renato Rios, Marlos Cortez, Marcel Miyake

**Affiliations:** ^1^ Department of Otolaryngology Santa Casa of São Paulo School of Medical Sciences São Paulo Brazil

**Keywords:** nasal lavage, nasal polyps, quality of life, sinusitis, steroids, sinus surgery

## Abstract

**Background:**

The indication for nasal irrigation with corticosteroids after sinus surgery in patients with Chronic Rhinosinusitis with Nasal Polyps (CRSwNP) is well established, as surgery facilitates distribution throughout the sinonasal cavity. However, it remains unknown whether this approach could also provide therapeutic benefit prior to surgery. This study aims to compare the effect of budesonide versus saline nasal irrigation in surgically naive CRSwNP patients.

**Methods:**

A randomized, double‐blind, placebo‐controlled parallel‐group study was conducted in 52 patients with diffuse type 2 CRSwNP with no previous sinus surgery. Patients were randomized to receive either 1 mg budesonide or saline nasal irrigation twice daily for four weeks. The primary outcome was the change in the 22‐item Sinonasal Outcome Test (SNOT‐22). Secondary outcomes included the Visual Analogue Scale (VAS), Nasal Polyp Score (NPS), and the Connecticut (CCCRC) olfactory test.

**Results:**

A total of 52 patients were randomized (mean age 50.1 ± 12.9 years; 51.9% female). The intention‐to‐treat (ITT) analysis showed that the budesonide nasal irrigation group demonstrated a significantly greater improvement in SNOT‐22 (mean difference: 18.1 points [95% CI: 3.4 to 32.8; *p =* 0.017]), VAS (mean difference 2.24 points [95% CI: 0.35 to 4.13; *p =* 0.018]) and NPS (mean difference: 0.73 points [95% CI: 0.25 to 1.21]; *p =* 0.003). No significant differences were observed between groups in CCCRC.

**Conclusion:**

Budesonide nasal irrigation may be an important tool for controlling sinonasal symptoms in patients with CRSwNP who are not candidates for sinus surgery or while awaiting surgical treatment.

## 1 Background

Chronic rhinosinusitis with nasal polyps (CRSwNP) is an inflammatory disease of the upper airways affecting approximately 2%–4% of the general population, associated with substantial morbidity and a marked negative impact on quality of life. Management is challenging due to high rates of refractoriness and recurrence [1, 2, 3].

Oral corticosteroids are considered the gold standard for medical treatment but are limited by systemic side effects, restricting their use to short courses [4]. Intranasal corticosteroid sprays, while safer, have limited efficacy in patients with extensive polyps because the small spray volume and low penetration fail to adequately reach polyp surfaces and the inflamed sinonasal mucosa [5]. Persistent symptoms despite medical therapy often lead to functional endoscopic sinus surgery (FESS) to remove polyps, restore sinonasal ventilation and facilitate topical steroid distribution [6].

High‐volume, low‐pressure nasal irrigation with corticosteroid diluted in isotonic saline is a well‐established adjunctive therapy after FESS. Surgery improves mucosal exposure, and the larger drug volume enhances sinonasal deposition and local bioavailability compared with sprays, allowing delivery to deeper recesses and polyp surfaces inaccessible to conventional devices. These advantages translate into improved symptom control and reduced recurrence without increasing systemic side effects, despite the higher administered dose. Theoretically, patients who did not respond to clinical treatment with intranasal corticosteroid spray and who would otherwise be referred for FESS could benefit from corticosteroid nasal irrigation, since the greater volume, dose, and distribution achieved through irrigation have been demonstrated in patients who have already undergone surgery; but this intervention is still poorly studied [7, 8, 9, 10]. Therefore, this study aims to compare the effect of budesonide versus saline nasal irrigation in surgically naïve CRSwNP patients, assessing short‐term symptom improvement through validated patient‐reported outcome measures and objective assessments.

## 2 Methods

### 2.1 Study Design and Participants

This was a randomized, double‐blind, placebo‐controlled clinical trial designed to compare the efficacy of isotonic saline nasal irrigation combined with budesonide (1 mg) versus placebo in patients with CRSwNP who had never undergone endoscopic sinus surgery. Participants were recruited from the outpatient clinic of the Department of Otolaryngology, Santa Casa de São Paulo, between 2019 and 2024. The study protocol was approved by the Institutional Review Board of Santa Casa de São Paulo (CAE 13229319.9.0000.5479), and written informed consent was obtained from all participants.

### 2.2 Inclusion and Exclusion Criteria

Patients included in the study had a diagnosis of primary diffuse CRSwNP, according to EPOS 2020 criteria [3], which encompasses the phenotypes of eosinophilic CRS (eCRS) and allergic which encompasses the phenotypes of eCRS and allergic fungal rhinosinusitis (AFRS). Although central compartment atopic disease (CCAD) is also classified as a type‐2 primary diffuse CRS, it was not included. Aspirin‐exacerbated respiratory disease (AERD), despite being considered a secondary CRS phenotype, was also included. Inclusion criteria included a minimum endoscopic nasal polyp score (NPS) of 2 in each nostril, a baseline Sino‐Nasal Outcome Test‐22 (SNOT‐22) score ≥30, and at least two of the following symptoms for a minimum of 12 weeks: Nasal obstruction or discharge (anterior or posterior), facial pain or pressure, and a reduced or lost sense of smell.

Exclusion criteria included prior endoscopic sinus surgery, previous treatment with biologics (anti‐IgE, anti‐IL‐4, anti‐IL‐5), current use of immunosuppressive therapy, antibiotic use within 4 weeks prior to enrollment, or sinonasal disease associated with systemic conditions such as immunodeficiency, Churg–Strauss vasculitis, granulomatosis with polyangiitis, autoimmune disease, or connective tissue disorders. Patients with anatomical abnormalities that could prevent effective nasal irrigation (obstructive septal deviation, choanal atresia, or complete nasal cavity obstruction by polyps), history of ocular herpes, pregnancy or breastfeeding, known hypersensitivity to budesonide, glaucoma, or use of systemic or topical nasal corticosteroids within 2 weeks prior to inclusion (or unwillingness to discontinue such medications) were also excluded. In addition, patients unable or unwilling to provide informed consent or comply with study procedures were not eligible.

Participants with asthma were allowed to continue their regular inhaled corticosteroid therapy; however, any patient requiring systemic corticosteroids during the study was withdrawn.

### 2.3 Intervention

Following eligibility confirmation and completion of a washout period—2 weeks for any intranasal corticosteroid sprays or 4 weeks for any systemic corticosteroids (oral, intravenous, or intramuscular)—participants were randomized (1:1) to the intervention group (budesonide) or the control group (placebo). Randomization was performed by a research pharmacy in blocks of 10, with variable block sizes, using a computer‐generated sequence. Only the pharmacist was aware of group allocation; participants and study staff remained blinded throughout.

The intervention group received glycerinated budesonide solution 1% (0.5 mg/per drop), while the control group received 5% glycerin solution identical in color, appearance, taste, and packaging. The nasal irrigation formulation composed of 5% glycerin and 1% budesonide is widely employed in clinical practice in Brazil, primarily due to its lower cost and greater availability compared to other commercial preparations [11]. Solutions were stored at controlled room temperature (15°C–30°C) in their original light‐protected containers. Each patient received a nasal irrigation kit containing one applicator bottle and 60 saline sachets. For preparation, participants were instructed to fill the applicator with 240 mL of filtered water, add one sachet and two drops of the assigned solution. Approximately half of the prepared volume (120 mL) was irrigated into each nostril twice daily for four weeks at home. The first dose of treatment was administered at the hospital under supervision to ensure correct technique and to verify that no nasal obstruction would interfere with the administration. All study medications and devices were provided free of charge.

Participants attended follow‐up visits at two and four weeks. At each visit, subjective and objective outcomes were collected and adverse events were assessed. The following outcome measures were obtained: SNOT‐22, “Connecticut Chemosensory Clinical Research Center” (CCCRC) smell test, visual analog scale (VAS), and NPS. Patients were permitted to miss no more than one dose per week to remain in the study.

### 2.4 Outcomes

The primary endpoint was the change in SNOT‐22 score from baseline to four weeks. The SNOT‐22 is a validated 22‐item questionnaire assessing sinonasal symptoms, with scores ranging from 0 to 110 (higher scores indicate greater severity) and a minimal clinically important difference (MCID) of 14 points [12].

Secondary endpoints included patient‐reported symptom severity using a Visual Analogue Scale (VAS; 0–10 cm) for global sinonasal symptoms [13]. Since there is currently no validated MCID for a global sinonasal VAS, we adopted a provisional threshold of 1 point on the 0–10 scale. This threshold represents approximately 10% of the total scale, consistent with established MCIDs for VAS instruments in other respiratory and symptom‐based conditions, and proportionally aligned with the validated SNOT‐22 MCID (∼11% of its total scale) [14, 15, 16]. Endoscopic NPS was assessed on a scale from 0 to 4 per nostril (0–8 total). Scoring was performed by two independent, blinded rhinologists using centrally reviewed endoscopy recordings. In addition to the numerical NPS, reviewers provided a subjective judgment of improvement between time points. A 1‐point change was considered the MCID, consistent with prior studies [17, 18, 19].

Finally, Lund–Mackay Score (LMS) was evaluated on sinus CT performed at the end of the intervention, scored by an independent blinded reviewer on a scale from 0 to 24, with higher scores indicating greater opacification [20].

### 2.5 Safety Assessment

Safety and tolerability were assessed by monitoring adverse events, vital signs, and physical examination at all study visits.

### 2.6 Statistical Analysis

All analyses followed the intention‐to‐treat principle. Missing data were imputed using last observation carried forward. Baseline characteristics were summarized descriptively. Normality of continuous outcomes was assessed with the Shapiro–Wilk test; as outcomes (SNOT‐22, VAS, NPS, CCCRC) were not normally distributed, between‐group comparisons of change from baseline to Week 4 were performed with the Mann–Whitney U test. The chi‐square test was used to compare the proportion of patients achieving the MCID. To assess whether demographic and clinical characteristics (age, sex, race, asthma, environmental allergy, aspirin sensitivity, profession, and smoking status) modified the effect of the intervention on SNOT‐22 scores, multivariable linear regression models were fitted for continuous outcomes and logistic regression models for binary outcomes. Each model included an interaction term between the treatment group and the covariate of interest. Interaction effects with *p* < 0.10 were considered indicative of potential effect modification. Sample size estimation was based on the SNOT‐22, using an MCID of 14 points derived from the Brazilian‐Portuguese validation [12], a standard deviation of 21.95, *α* = 0.05 and 80% power. Considering a 20% dropout rate, a minimum of 26 patients per group was required [7, 21]. Analyses were performed in SPSS v26 (IBM, Armonk, NY), with *p *< 0.05 considered significant, except for interaction terms as noted above.

## 3 Results

### 3.1 Participants

**FIGURE 1:**
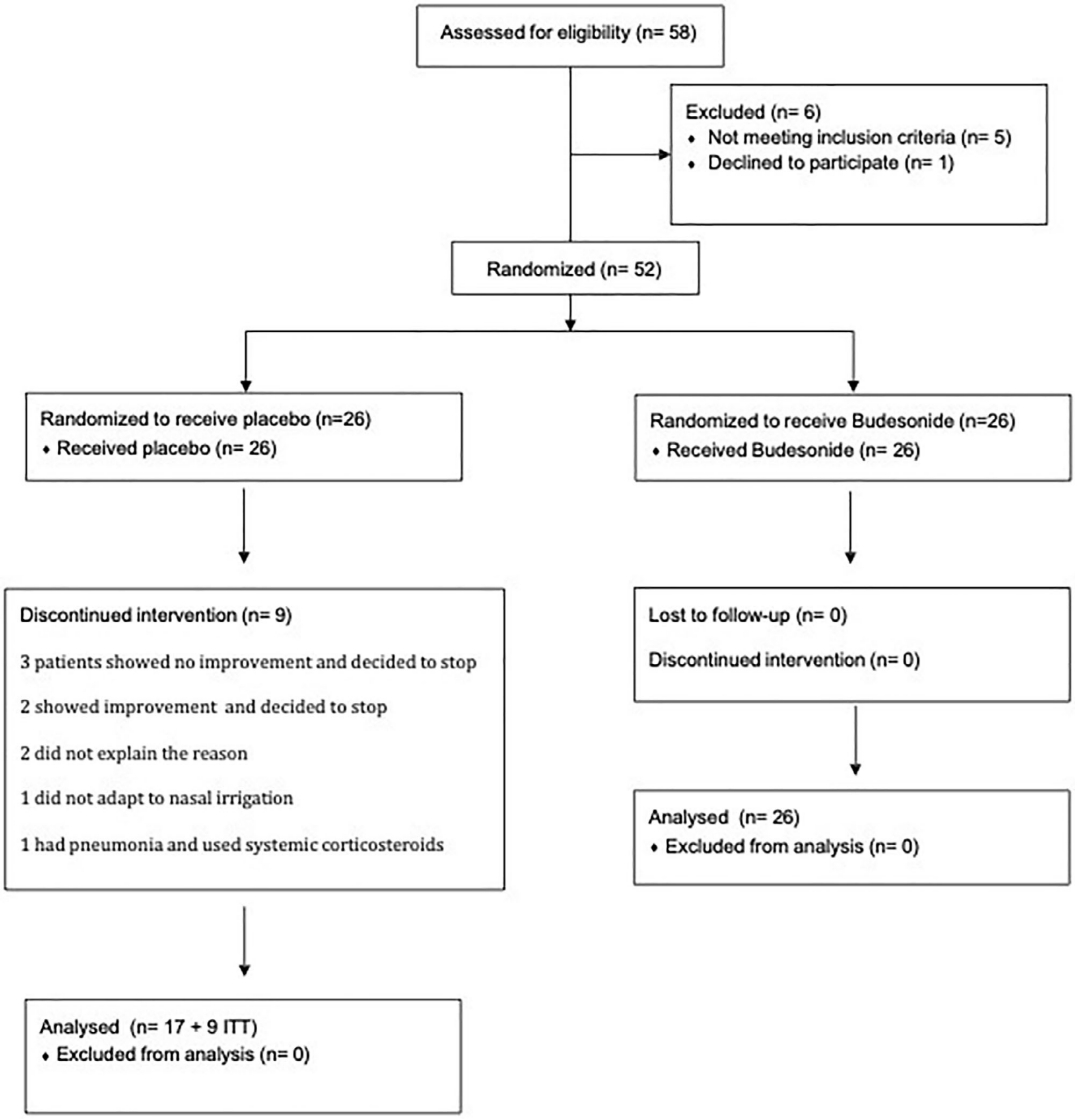
Patients enrolled and included in the analysis.

Overall, 58 patients were recruited, of whom 52 underwent randomization (Figure 1). A total of 43 patients completed the study — 26 in the budesonide group and 17 in the placebo group. All patients were actively questioned about the use of rescue corticosteroids throughout the entire follow‐up period, and no patient reported having used them. Demographic and baseline clinical characteristics were generally comparable between groups (Table 1).

**TABLE 1:** Baseline demographic and clinical characteristics of patients in the placebo and budesonide groups.

	Placebo (*n* = 26)	Budesonide (*n* = 26)
Age, mean (SD)	46,5 (12.1)	53,8 (13.7)
Male sex, No. (%)	11 (42.3)	14 (53.8)
White race, No. (%)	12 (46.2)	11 (42.3)
African American race, No. (%)	5 (19.2)	4 (15.4)
Asian race, No. (%)	0 (0)	1 (3.8)
Asthma, No. (%)	13 (50.0)	10 (38.5)
Environmental allergy, No. (%)	16 (61.5)	18 (69.2)
Aspirin sensitivity, No. (%)	2 (7,6)	3 (11.5)
Profession with exposure to toxic aerosols, No. (%)	8 (30.8)	7 (26.9)
Smoking, No. (%)	3 (11.5)	3 (11.5)

*Note*: Data are presented as mean (standard deviation) for continuous variables and number (percentage) for categorical variables.

### 3.2 Primary End Point (Table 2)

#### 3.2.1 Sinonasal Outcome Test (SNOT‐22)

**FIGURE 2:**
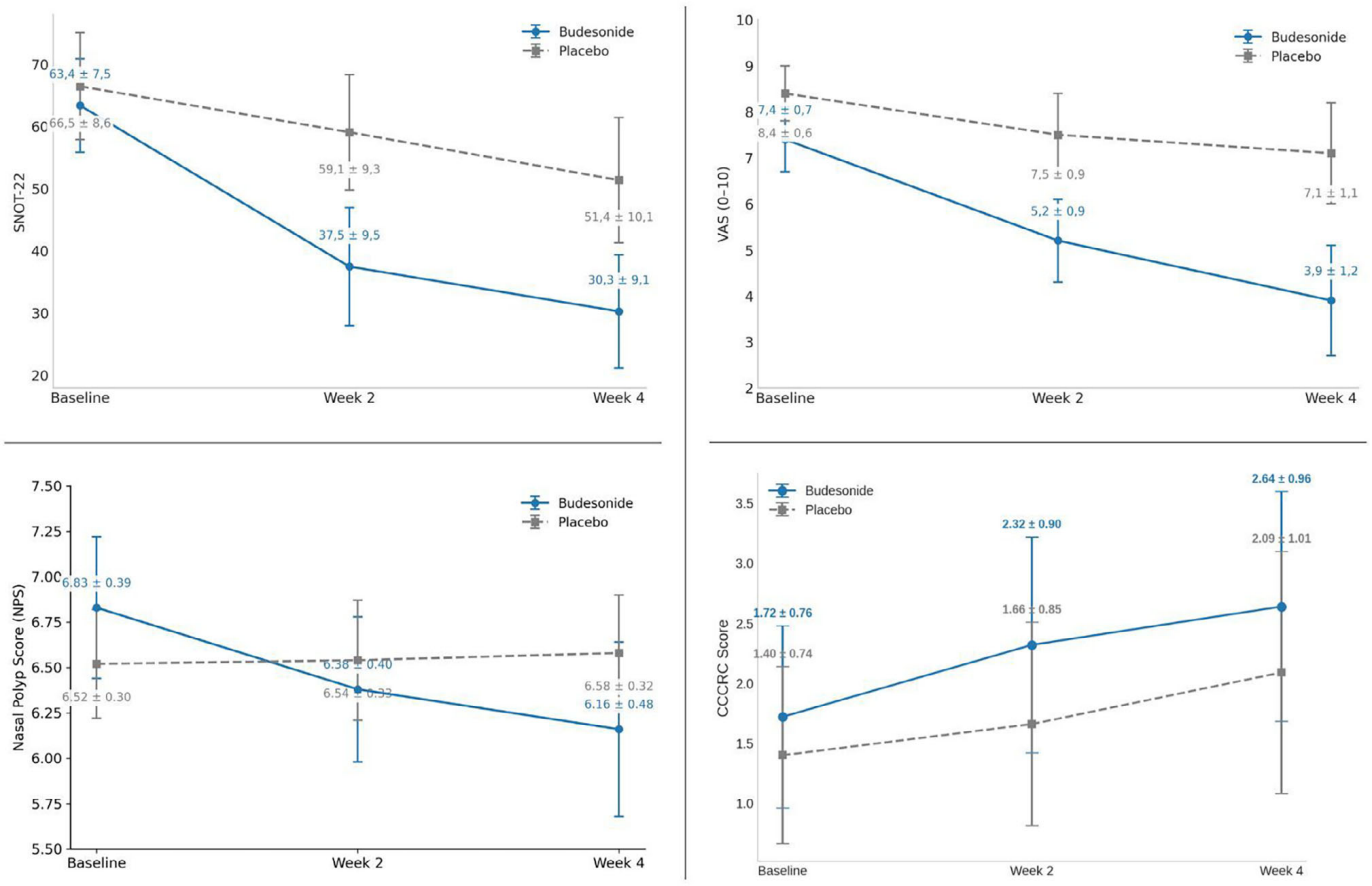
Primary and secondary end points for all patients. The *p‐*value reflects the comparison of mean changes between groups from baseline to week 4. Error Bars indicates the 95% CI.

After 4 weeks, the intention‐to‐treat analysis showed a mean improvement of −33.2 points [95% CI: −38.7 to −27.6] in the budesonide group and −15.1 points [95% CI: −20.0 to −10.2] in the placebo group (Figure 2). The between‐group difference was 18.1 points [95% CI: 3.4 to 32.8; *p* = 0.017], significantly favoring budesonide. Clinically meaningful improvement, defined as exceeding the MCID, was observed in 19 of 26 patients (73.1%) in the budesonide group and 8 of 26 (30.8%) in the placebo group (*p* = 0.049).

**TABLE 2:** Outcomes of primary and secondary endpoints in patients with chronic rhinosinusitis with nasal polyps (CRSwNP) treated with budesonide nasal irrigation versus placebo.

	Budesonide (*n* = 26)			Placebo (*n* = 26)				
	Baseline, Mean (SD)	Week 4, Mean (SD)	Absolute Change From Baseline, LS Mean (95% CI)	Baseline, Mean (SD)	Week 4, Mean (SD)	Absolute Change From Baseline, LS Mean (95% CI)	**Absolute Difference for Budesonide vs. Control, LS Mean (95% CI)**	***p‐*value**
Primary End Point								
SNOT 22	63.4 (19.5)	30.3 (23.7)	−33.15 (−44.84 to −21.47)	66.5 (22.3)	51.4 (26.3)	−15.08 (−25.20 to −4.95)	**18.07 (3.37 to 32.77)**	**0.020**
Secundary End Points								
VAS	7.43 (1.81)	3.9 (3.02)	−3.52 (−5.11 to −1.41)	8.36 (1.66)	7.07 (2.88)	−1.28 (−2.48 to −0.97)	**−2.23 (−4.12 to −0.35)**	**0.018**
NPS	6.83 (1.01)	6.16 (1.24)	−0.67 (−1.09 to −0.25)	6.52 (0.78)	6.58 (0.83)	0.06 (−0.23 to 0.35)	**−0.73 (−1.21 to −0.25)**	**0.003**
CCCRC	1.72 (1.99)	2.64 (2.49)	0.92 (0.23 to 1.62)	1.40 (1.93)	2.09 (2.63)	0.68 (−0.06 to 1.42)	**0.24 (−1.20 to 0.72)**	**0.565**
LMS^a^	—	15.7 (2.4)	—	—	12.8 (2.4)	—	**2.90 (−2.04 to 7.84)**	**0.250**

*Note*: Values are expressed as mean difference (95% CI). Bolded values indicates statistically significant differences between the budesonide and placebo groups.

Abbreviations: CCCRC, Connecticut Chemosensory Clinical Research Center test; LMS, Lund–Mackay Score; SNOT‐22, Sino‐Nasal Outcome Test‐22; VAS, Visual Analog Scale.

^a^ Computed tomography of the paranasal sinuses performed after 4 weeks of intervention; 23 patients in the budesonide group and 14 in the placebo group).

### 3.3 Secondary End Points (Table 2)

#### 3.3.1 Visual Analog Scale

Patients treated with budesonide also showed greater benefit on VAS scores. After 4 weeks, mean improvement was −3.53 points [95% CI: −5.1 to −1.94] in the budesonide group compared with −1.29 points [95% CI: −2.48 to −0.97] in the placebo group, yielding a between‐group difference of 2.24 points [95% CI: 0.352 to 4.13; *p* = 0.018] (Figure 2). Clinically significant improvement beyond the MCID was achieved by 20 of 26 patients (76.9%) in the budesonide group and 13 of 26 (50.0%) in the placebo group (*p* = 0.044).

#### 3.3.2 Nasal Polyp Score

Inter‐rater reliability was strong (*κ* = 0.805; *p *< 0.001). After 4 weeks, mean reduction in NPS was −0.67 points [95% CI: −0.92 to −0.42] in the budesonide group versus −0.06 points [95% CI: −0.33 to 0.21] in the placebo group. The mean between‐group difference was 0.73 points [95% CI: 0.25 to 1.21; *p* = 0.003], confirming a statistically and clinically significant superiority of budesonide over placebo (Figure 2). Clinically relevant improvement was observed in 20 of 26 patients (76.9%) in the budesonide group compared with 13 of 26 (50.0%) in the placebo group (*p* = 0.04).

#### 3.3.3 CCCRC Test

Changes in olfactory function, as measured by the CCCRC test, did not differ significantly between groups. At 4 weeks, the mean improvement was +0.58 points [95% CI: −0.12 to 1.28] in the budesonide group compared with +0.34 points [95% CI: −0.41 to 1.09] in the placebo group, resulting in a between‐group difference of 0.24 points [95% CI: −0.72 to 1.20; *p* = 0.63] (Figure 2). While the difference was not statistically significant, a higher proportion of patients in the budesonide group achieved improvement exceeding the MCID, 11 of 26 (42.3%) versus 6 of 26 (23.1%) in the placebo group (*p* = 0.139).

#### 3.3.4 Lund–Mackay Score

At the end of the 4‐week treatment period, 23 patients in the budesonide group underwent computed tomography, with a mean Lund–Mackay score of 15.7 [SD 2.4]. In the placebo group, 14 patients were evaluated, showing a mean score of 12.8 [SD 2.4]. The mean between‐ group difference was 2.9 points [95% CI, −2.04 to 7.84; *p* = 0.25], suggesting greater disease severity in the budesonide group.

#### 3.3.5 Interaction Analysis

No significant interactions were observed between SNOT‐22 outcomes and any of the demographic or clinical characteristics evaluated (age, sex, race, asthma, environmental allergy, aspirin sensitivity, profession, or smoking status).

### 3.4 Safety

The frequency of adverse events did not differ between groups (Table 3). Headache was the most frequently reported adverse event in the budesonide group (5 vs. 1 in the placebo group). In addition, one patient experienced a self‐limited episode of epistaxis. However, these differences were not statistically significant, and no treatment discontinuations were required.

**TABLE 3:** Adverse events reported in the placebo and budesonide groups.

	Placebo (*n* = 26)	Budesonide (*n* = 26)
**Adverse event, No. (%)**	14 (53.8)	15 (57.7)
Epistaxis, No. (%)	0 (0)	1 (3.8)
Cough, No. (%)	2 (7.7)	2 (7.7)
Otalgia, No. (%)	3 (11.5)	3 (11.5)
Skin reactions, No. (%)	0 (0)	2 (7.7)
Nausea, No. (%)	1 (3.8)	1 (3.8)
Tachycardia, No. (%)	1 (3.8)	0 (0)
Nasal irritation, No. (%)	2 (7.7)	2 (7.7)
Headache, No. (%)	1 (3.8)	5 (19.2)
Others, No. (%)	7 (26.9)	6 (23.1)

*Note*: Data are presented as number (percentage).

### 3.5 Discussion

This is the first double‐blinded placebo controlled clinical trial to evaluate the efficacy nasal irrigation with budesonide in patients with diffuse CRSwNP who had not undergone FESS. Budesonide irrigation demonstrated superiority over placebo in improving sinonasal symptoms, as evidenced by significant reductions in SNOT‐ 22 and VAS scores after 4 weeks of treatment, as well as a reduction in polyp size. This improvement was observed both in the mean differences between groups and in the proportion of patients achieving an MCID. These findings support the concept that corticosteroid irrigations are effective not only in postoperative patients with wide paranasal sinus exposure, as previously established in the literature, but also as a therapeutic option for surgically naïve patients with CRSwNP [7, 8, 10]

Adherence to treatment was excellent in the budesonide group, with no dropouts, whereas nine patients in the placebo arm discontinued due to lack of response. These findings underscore not only the clinical efficacy but also the tolerability and acceptance of budesonide irrigation. Safety outcomes were consistent with previous reports, with adverse events generally mild, transient, and comparable between groups [8, 20, 21].

The interaction analyses indicated that demographic and clinical characteristics, including polyp size, asthma, and aspirin intolerance, did not significantly modify the treatment effect. This suggests that the benefits of budesonide nasal irrigation are consistent across patient subgroups, supporting its broad applicability in clinical settings.

Importantly, the study population was homogeneous, including only patients with moderate‐ to‐severe CRSwNP without prior FESS, thereby overcoming limitations of previous trials that enrolled heterogeneous populations, such as those combining patients with CRS with and without polyps, or with and without previous sinus surgery [8, 10, 17, 20]. Moreover, it is noteworthy that, although AFRS was included among the eligibility criteria, no patients with this condition were screened or enrolled in the study, reinforcing the homogeneity of the analyzed population.

The nasal irrigation formulation composed of 5% glycerin and 1% budesonide is widely employed in clinical practice in Brazil, primarily due to its lower cost and greater availability compared to other commercial preparations. This characteristic supports its adoption as a viable therapeutic alternative in different healthcare settings, particularly in contexts where accessibility is a determining factor for treatment adherence [11].

Several limitations should be acknowledged. The relatively short follow‐up period (4 weeks) prevents conclusions regarding the durability of symptom improvement or long‐term reduction in surgical requirements. The post‐treatment CT scores (Lund–Mackay) in the budesonide group were slightly higher than those in the control group. Since pre‐intervention imaging was not performed for ethical reasons, it is not possible to determine whether this difference was already present at baseline. Nevertheless, the higher LMS score in the budesonide group should not be interpreted as treatment‐related worsening, as there were no dropouts in this group and the clinical outcomes — SNOT‐22, VAS, and NPS — showed greater improvement with budesonide than with placebo. Therefore, the radiological difference may reflect unmeasured baseline disparities or selective attrition in the placebo group, rather than a negative effect of the intervention. Finally, we used the value of 1 point as the MCID for the VAS because it represents a provisional threshold, supported by previous methodological studies and extrapolations from related instruments, given that no formally established MCID exists for global VAS in CRSwNP. Importantly, the improvements observed in our study exceeded this threshold by a wide margin, reinforcing the clinical relevance of the effects found. Despite these limitations, the study has important clinical implications. Many patients with CRSwNP face barriers to surgery—whether due to medical contraindications, socioeconomic factors, or long waiting times. Budesonide nasal irrigation represents a safe, effective, and accessible alternative for symptom control in these scenarios. By demonstrating rapid and meaningful symptom improvement, this intervention may reduce patient burden while awaiting surgical treatment and potentially delay or reduce the need for more invasive procedures [3, 22].

## 4 Conclusion

Budesonide nasal irrigation is an effective, well‐tolerated therapy for non‐operated CRSwNP patients, improving quality of life and reducing polyp size. These findings support its use as a practical and evidence‐based option in both primary management and in patients for whom surgery is not immediately feasible.

## Funding

The authors have nothing to report.

## Conflicts of Interest

Miyake MM reports consulting fees from Myralis Farmacêutica and honoraria for lectures from Myralis Farmacêutica, GlaxoSmithKline, Sanofi, Pfizer, and AstraZeneca.

## References

[1] C. Bachert , B. Marple , R. J. Schlosser , et al., “Adult Chronic Rhinosinusitis,” Nature Reviews Disease Primers 6, no. 1 (2020): 86, 10.1038/s41572-020-00218-1. 33122665

[2] W. W. Stevens , R. P. Schleimer , and R. C. Kern , “Chronic Rhinosinusitis With Nasal Polyps,” Journal of Allergy and Clinical Immunology: In Practice 4, no. 4 (2016): 565–572, 10.1016/j.jaip.2016.04.012. 27393770 PMC4939220

[3] W. J. Fokkens , V. J. Lund , C. Hopkins , et al., “European Position Paper on Rhinosinusitis and Nasal Polyps 2020,” Rhinology 58 (2020): 1–464. 10.4193/Rhin20.600 32077450

[4] D. B. Allen , “Systemic Effects of Intranasal Steroids: An Endocrinologist's Perspective,” Journal of Allergy and Clinical Immunology 106, no. 4 SUPPL (2000): S179–S190, 10.1067/mai.2000.110038. 11032642

[5] K. L. Ah‐See , J. MacKenzie , and K. W. Ah‐See , “Management of Chronic Rhinosinusitis,” BMJ 345, no. 7881 (2012): e7054, 10.1136/bmj.e7054. 23111434

[6] R. J. Harvey , J. C. Goddard , S. K. Wise , and R. J. Schlosser , “Effects of Endoscopic Sinus Surgery and Delivery Device on Cadaver Sinus Irrigation,” Otolaryngology—Head and Neck Surgery 139, no. 1 (2008): 137–142, 10.1016/j.otohns.2008.04.020. 18585576

[7] R. J. Harvey , K. Snidvongs , L. H. Kalish , G. M. Oakley , and R. Sacks , “Corticosteroid Nasal Irrigations Are More Effective Than Simple Sprays in a Randomized Double‐Blinded Placebo‐Controlled Trial for Chronic Rhinosinusitis After Sinus Surgery,” International Forum of Allergy & Rhinology 8, no. 4 (2018): 461–470, 10.1002/alr.22093. 29394004

[8] S. Tait , D. Kallogjeri , J. Suko , S. Kukuljan , J. Schneider , and J. F. Piccirillo , “Effect of Budesonide Added to Large‐Volume, Low‐Pressure Saline Sinus Irrigation for Chronic Rhinosinusitis a Randomized Clinical Trial,” JAMA Otolaryngology—Head & Neck Surgery 144, no. 7 (2018): 605–612, 10.1001/jamaoto.2018.0667. 29879268 PMC6145785

[9] H. Salati , N. Singh , M. Khamooshi , S. Vahaji , D. F. Fletcher , and K. Inthavong , “Nasal Irrigation Delivery in Three Post‐FESS Models From a Squeeze‐Bottle Using CFD,” Pharmaceutical Research 39, no. 10 (2022): 2569–2584, 10.1007/s11095-022-03375-y. 36056272 PMC9556402

[10] S. Ahamed , D. Samson , R. Sundaresan , B. Balasubramanya , and R. Thomas , “Double Blinded Randomized Controlled Trial Comparing Budesonide and Saline Nasal Rinses in the Post‐Operative Management of Chronic Rhinosinusitis,” Indian Journal of Otolaryngology and Head and Neck Surgery 76, no. 1 (2024): 408–413, 10.1007/s12070-023-04173-7. 38440477 PMC10909031

[11] G. R. Luz‐Matsumoto , E. Cabernite‐Marchetti , and L. S. K. Sasaki , “Nasal Irrigation With Corticosteroids in Brazil: The Clinical Response of 1% Compounded Budesonide Drops and Betamethasone Cream,” Brazilian Journal of Otorhinolaryngology 88 (2022): S32–S41, 10.1016/j.bjorl.2021.06.008. 34563470 PMC9800950

[12] E. M. Kosugi , V. G. Chen , V. M. Guerreiro Da Fonseca , et al., “Translation, Cross‐Cultural Adaptation and Validation of SinoNasal Outcome Test (SNOT)‐22 to Brazilian Portuguese Abstract,” Brazilian Journal of Otorhinolaryngology 77, no. 5 (2011): 663–669, http://www.bjorl.org/http://www.bjorl.org/. 22030978 10.1590/S1808-86942011000500021 PMC9443728

[13] S. B. Bird and E. W. Dickson , “Clinically Significant Changes in Pain Along the Visual Analog Scale,” Annals of Emergency Medicine 38, no. 6 (2001): 639–643, 10.1067/mem.2001.118012. 11719742

[14] M. L. Barnes , S. Vaidyanathan , P. A. Williamson , and B. J. Lipworth , “The Minimal Clinically Important Difference in Allergic Rhinitis,” Clinical and Experimental Allergy 40, no. 2 (2010): 242–250, 10.1111/j.1365-2222.2009.03381.x. 19895590

[15] V. H. Shih , A. F. Slagle , C. Ivanescu , et al., “Psychometric Validation and Meaningful Change Thresholds of the New Nasal Polyposis Symptom Diary,” Annals of Otology, Rhinology and Laryngology 132, no. 12 (2023): 1638–1648, 10.1177/00034894231177769. 37271980 PMC10571433

[16] L. P. Hoehle , K. M. Phillips , M. M. Speth , D. S. Caradonna , S. T. Gray , and A. R. Sedaghat , “Responsiveness and Minimal Clinically Important Difference for the EQ‐5D in Chronic Rhinosinusitis,” Rhinology 57, no. 2 (2019): 110–116, 10.4193/Rhin18.122. 30175337

[17] S. S. Jeong , T. Chen , S. A. Nguyen , T. S. Edwards , and R. J. Schlosser , “Correlation of Polyp Grading Scales With Patient Symptom Scores and Olfaction in Chronic Rhinosinusitis: A Systematic Review and Meta‐Analysis,” Rhinology 60, no. 5 (2022): 322–334, 10.4193/Rhin22.011. 36191585

[18] V. J. Lund' and I. S. Mackay , “Staging in Rhinosinusitus,” Rhinology 31 (1993): 183–184. 8140385

[19] J. Braid , L. Islam , C. Gugiu , T. A. Omachi , and H. Doll , “Meaningful Changes for Efficacy Outcomes in Patients With Chronic Rhinosinusitis With Nasal Polyps,” World Allergy Organization Journal 16, no. 5 (2023): 100776, 10.1016/j.waojou.2023.100776. 37214171 PMC10197100

[20] P. Jiramongkolchai , A. Peterson , D. Kallogjeri , et al., “Randomized Clinical Trial to Evaluate mometasone Lavage vs Spray for Patients With Chronic Rhinosinusitis Without Nasal Polyps Who Have Not Undergone Sinus Surgery,” International Forum of Allergy & Rhinology 10, no. 8 (2020): 936–943, 10.1002/alr.22586. 32470217 PMC8932402

[21] K. Snidvongs , E. Pratt , D. Chin , R. Sacks , P. Earls , and R. J. Harvey , “Corticosteroid Nasal Irrigations After Endoscopic Sinus Surgery in the Management of Chronic Rhinosinusitis,” International Forum of Allergy & Rhinology 2, no. 5 (2012): 415–421, 10.1002/alr.21047. 22566474

[22] K. Matos De Araujo , S. Junior , S. Tomita , A. Octavio , and A. Kos , “The Problem of Waiting Lines for Otorhinolaryngology Surgeries in Public Services,” Brazilian Journal of Otorhinolaryngology 71 (2005): 256–262, http://www.rborl.org.br/. 16446927 10.1016/S1808-8694(15)31321-5 PMC9450532

